# Placebo and other psychological interactions in headache treatment

**DOI:** 10.1007/s10194-012-0422-0

**Published:** 2012-02-26

**Authors:** A. Autret, D. Valade, S. Debiais

**Affiliations:** 1 CHRU TOURS, Tours, France; 2 Centre d′Urgences céphalées, Hôpital Lariboisière, Paris, France

**Keywords:** Migraine, Placebo, Headache treatment

## Abstract

We present a theory according which a headache treatment acts through a specific biological effect (when it exists), a placebo effect linked to both expectancy and repetition of its administration (conditioning), and a non-specific psychological effect. The respective part of these components varies with the treatments and the clinical situations. During antiquity, suggestions and beliefs were the mainstays of headache treatment. The word placebo appeared at the beginning of the eighteenth century. Controversies about its effect came from an excessive interpretation due to methodological bias, inadequate consideration of the variation of the measure (regression to the mean) and of the natural course of the disease. Several powerful studies on placebo effect showed that the nature of the treatment, the associated announce, the patients’ expectancy, and the repetition of the procedures are of paramount importance. The placebo expectancy is associated with an activation of pre-frontal, anterior cingular, accumbens, and periacqueducal grey opioidergic neurons possibly triggered by the dopaminergic meso-limbic system. In randomized control trials, several arms design could theoretically give information concerning the respective part of the different component of the outcome and control the natural course of the disease. However, for migraine and tension type headache attacks treatment, no three arm (verum, placebo, and natural course) trial is available in the literature. Indirect evidence of a placebo effect in migraine attack treatment, comes from the high amplitude of the improvement observed in the placebo arms (28% of the patients). This figure is lower (6%) when using the harder criterium of pain free at 2 h. But these data disregard the effect of the natural course. For prophylactic treatment with oral medication, the trials performed in the last decades report an improvement in 21% of the patients in the placebo arms. However, in these studies the duration of administration was limited, the control of attacks uncertain as well as the evolution of the co-morbid psycho-pathology. Considering the reviews and meta-analysis of complex prophylactic procedures, it must be concluded that their effect is mostly linked to a placebo and non-specific psychological effects. Acupuncture may have a slight specific effect on tension type headache, but not on migraine. Manual therapy studies do not exhibit difference between manipulation, mobilization, and controls; touch has no proven specific effect. A comprehensive efficacy review of biofeedback studies concludes to a small specific effect on tension type headache but not on migraine. A review of behavioral treatment conclude to an interesting mean improvement but did not demonstrated a specific effect with the exception of a four arm study including a pseudo meditation control group. Expectation-linked placebo, conditioning, and non-specific psychological effects vary according clinical situations and psychological context; likely low in RCT, high after anempathic medical contact, and at its maximum with a desired charismatic healer. The announcements of doctors strongly influence the beliefs of patients, and in consequence their pain and anxiety sensibilities; this modulates the amplitude of the placebo and the non-specific psychological effects and is therefore a major determinant of the therapeutic success. Furthermore, any repetitive contact, even through a placebo, may interfere positively with the psychopathological co-morbidity. One has to keep in mind that the non-specific psychological interactions play a major role in the improvement of the majority of the headache sufferers.

## Introduction

In pre-scientific civilizations, suggestions of the healer and the beliefs of the patients were the mainstays of headache treatment: in the Ebers papyrus [1], which was written in approximately 1200 BC, one treatment was to firmly bind a clay crocodile holding a grain in its mouth to the patient’s head with a strip of linen bearing the names of the gods. Another was to use a bandage with honey and coriander. In the dialogues of Plato (Charmides), approximately 400 BC, Socrates states that he used a leaf and a charm to treat headache.

The aim of this review is to determine from studies currently available in the PubMed database (US National Library of Medicine, National Daily Institutes of Health), how placebo and psychological effects take their place beside the biological specific action of treatments in the headache management. To assess this issue, we present successively a review of the basic studies on placebo effect, the contribution of the randomized control trials (RTC) in headache, and a sketch of a theory on headache treatment healing in clinical practice, with a special regard on the psychological interactions.

## Some basic considerations concerning placebo

Interestingly, one had to wait until Hooper’s English medical dictionary in 1811 to see in the medical literature, the word “placebo”. It was used to designate a medicine given more to please than to treat (see [2]). The placebo is therefore, basically, an inert product which has to be compared to the active principle called verum; by extension, a complex therapeutic procedure has also to be compared to a sham (placebo) procedure. The scientific debate about placebo in therapeutic trials began in 1955 in a seminal article by Beecher [3] entitled “The powerful placebo”. This “power” remains controversial due to the concomitant confusion factors which have been comprehensively reviewed [4] (natural course of the disease, additional treatment, observer bias, irrelevant response, verum toxicity, patient bias, misquotation…) [5–7]. As we shall see below these misleading factors have to be controlled by an appropriated methodology. At the turn of the last century, a bio-psychological approach with several powerful studies shed light on the placebo phenomena [8].

### Influence of what is offer to the patients

The color and number of pills influence the outcome [9, 10]: placebo tablets given to students and told to have a psychological effect act as stimulants when they are red and depressants when blue, and two placebo pills have a higher effect than one. The branding increases the placebo effect [11]. Parenteral or subcutaneous administration is more efficient than oral administration [12, 13]. A pseudo-acupuncture sham device had a greater effect than a placebo pill in chronic arm pain [14]. Finally, the more complex the procedure including rituals, mysterious powers, high technology and surgery, the larger the effects that are seen [13, 14].

The associated announcement is of paramount importance. A placebo cream presented as a powerful local anesthetic only induces an effect where it is applied [15]. The amplitude of the analgesic effect of saline given as a pain-killer after thoracic surgery depends on previously announced analgesic power [16]. In healthy young adults, an exercise program induces psychological well-being only in the group where this psychological effect has been announced and emphasized [17].

Practitioners’ attitude influences the placebo effect: in irritable bowel syndrome treated by pseudo-acupuncture, a warm empathic interaction enhances the placebo effect, but only in patients with an high extraversion profile [18].

### Patients’ expectations and personality

The desire to be relieved, the expectations from the procedure, the memory of previous effects [19, 20], and the overt and covert meanings of the procedure [8] influence the placebo effect according to an expectation response theory in which expectations are the major determinant of what will occur in a given situation [21].

However, a relationship between the placebo reactivity and a given psychological or a socio-cultural state was not evidenced in a large study on patients with a persistent distal upper arm pain; the dimensions tested were: anxiety, depression, belief in alternative medicine, sex, color (white/non-white), educational level, and age [22].

### Genuine placebo effect and confusion factors

The placebo effect linked to expectation is, therefore, a consequence of the idea of having received the verum. This genuine placebo effects have to be differentiated from the contingent events and from the natural course of the disease during the same period. In RCT, patients are often included when their symptoms worsen above a certain threshold. This threshold can be misleadingly reached when a fluctuating symptom is at its maximum or when it is erroneously measured in excess, thus a decrease or regression to the mean of the symptoms may have no biological signification (see review in [23]). Accordingly, a placebo effect can only be measured when comparing patients treated with placebo to non-treated patients during the same time period [7]. Even in this case, a bias can occur in the non-treated group due to the negative impact of lack of treatment.

### Neurobiology of the placebo effects associated with expectations

The first step was the discovery of a link between expectation, placebo improvement and opioidergic mediation. Indeed, after molar extraction, naloxone, a μ-opioid receptor antagonist, reduced the beneficial effect of saline that was presented as an analgesic [24].

Pioneering work in PET and in fMRI showed that the expectation linked placebo analgesia was associated with increased activity in the rostral anterior cingulum [25, 26]. Using PET measurements of the fixation potential of 11C-carfentanil, a μ-opioid receptor ligand, several authors [27, 28] showed the implication of an opioidergic network involving pre-frontal and anterior cingular cortex, accumbens nuclei and peri-acqueducal gray in expectation linked placebo effect. The meso-limbic and orbito-frontal dopaminergic systems has also been demonstrated to be implicated by simultaneously measuring the fixation potential of 11C-raclopride, a D2/D3 agonist. The placebo-induced expectation is associated with a bilateral increase in dopaminergic activity in the ventral putamen and nucleus accumbens, which seem to play a trigger role in μ-opioidergic activation. Furthermore, it has been shown that an increase in pain sensation (i.e., a nocebo effect) is associated with opioid and dopaminergic deactivation [29]. Finally, μ-opioidergic and D2/D3 dopaminergic systems are activated to different degrees, depending on the patient’s positive (placebo) or negative (nocebo) expectation [30]. The analgesic placebo-induced expectation network is a part of a wider emotion control system [31].

### Conditioning

A conditioning effect, not linked to expectations has been demonstrated. A study by Pavlov showed that, after coupling a tone (neutral stimulus) and apomorphine (unconditioned stimulus), the drug-induced symptoms still continue only after sound alone. There are several animal studies demonstrating that saline can induce an effect when replacing a drug given before repeatedly (see review in [32]). Such a conditioning has been demonstrated in humans, using the tourniquet paradigm (measuring daily the duration of hand contraction under ischemia); indeed ketorolac administered repeatedly reduced the pain sensation; then its substitution by a placebo, presented as an antibiotic was associated with the persistence of an analgesic effect; this effect persisted when the placebo was associated with naloxone [33]: this interesting experience demonstrates that in human, a conditioning may induce an analgesia which is not linked to an expectation, nor to an opioidergic mechanism.

When a treatment is given repeatedly, conditioning and expectation are intermingled, and the effect is reinforced with the length of the symptom-free period [34]. In an interesting study, conditioning was revealed to have a more powerful analgesic effect than expectation [32].

## Lessons given by RCT

We assume that the outcome of headache management is the result of additive actions of (1) a specific effect on headache mechanisms, (2) a placebo effect linked to the idea of having received the verum, and (3) a non-specific psychological covert intervention (empathy, kind listening, etc…) which can be at its maximum in some complex therapeutic procedures as acupuncture, touch and manual therapy, biofeedback.

Therefore, to demonstrate a genuine placebo effect for an oral treatment, one should ideally use, at least, a three arm trial design (verum, placebo, and no treatment) [6]. Differences between the verum and placebo reflect the specific effect. Differences between the placebo and no treatment measure the placebo effect. To analyze the complex procedure effect one should, also, control the non-specific psychological covert intervention by the mean of a pertinent “psychological” control group. Bias may come from a non-convincing sham procedure, or from the negative effect of being included in a “psychological”, or in a non-treated control group.

At this point, it appears interesting to clarify the relationships between placebo, non specific psychological intervention and psychotherapy [35]. The three act through psychological processes. Placebo effect is simply mostly based on an expectation after the announcement of given therapy and after conditioning, the non-specific psychological intervention is limited to empathy, kind listening without psychological base, and psychotherapy acts mostly through specific intentionally delivered psychological interactions.

### Acute treatment for headache attacks

No three arm trials have studied acute treatment [36]. In migraine, meta-analysis [37–42] of placebo arms show that in adult patients, at 2 h, a two point improvement (using a 0–3 scale) is seen in about 28–29% of patients and a pain-free state in 6–9% (verum 58% for improvement and of 29% for pain-free), however, with a high heterogeneity [40]. These figures demonstrate the specific effect of the verum included in the meta-analysis, and their amplitude suggests that a genuine placebo effect does exist, mostly when a permissive outcome is chosen. A high placebo efficacy is reported in children: meta-analysis of 13 studies found that at 2 h after administration, improvement was seen in 33% (23–43%) of children, and a pain-free state in 14% (9–18%) [43].

A meta-analysis of 37 studies about the treatment of tension type headache attacks shows that NSAIDs and acetaminophen have a significant specific effect [44], but not data are available to analyze a putative placebo effect.

### Prophylactic treatment and placebo effect

From a comprehensive meta-analysis of three arm trials [36], only five studied headache exclusively [45–49]; unfortunately, none of these studies considered the actual IHS classification. Only one three arm trial concerned oral medication, given 2 weeks, and did not evidence for a difference in headache score between the placebo an no-treatment [47].

In a comprehensive meta-analysis of oral prophylactic treatments RCT of migraine (32 studies) [50], the percentage of patients presenting a 50% reduction in the number of days with headache reported in the placebo arms is 21% (13–28%). There was a significant heterogeneity. The corresponding data for the verum was 41% (33–49%). The improvement under placebo was greater in parallel compared to cross-over studies and in European compared to North American trials. These studies were performed between 1998 and 2004, usually lasted 12 weeks and did not mention the level of control of the acute attacks. A recent follow-up during 16 months of migraine patients with an optimized attack treatment [51] showed an improvement with time without differences between placebo alone, beta blockers alone, or placebo associated with behavioral management, suggesting that the administration of any treatment, even a placebo, is sufficient to achieve an apparent therapeutic success [51].

In conclusion, a specific effect of the prophylactic oral treatments included in the meta-analysis seems to be demonstrated [52] only for a relatively short time use. A prophylactic effect of placebo is also suggested [50] for a short time period by the amplitude of the improvement observed in the placebo arm. Finally, one study suggests a placebo prophylactic efficacy for a long period [51].

Meta-analysis of studies of tension type headache prophylaxis with oral treatment provide conflicting results: a lack of superiority of antidepressant medication or myorelaxants over placebo is reported in one study [53], and a beneficial effect of tricyclic antidepressants in two others [54, 55]. No data are presented to evaluate a putative placebo effect. Interestingly, the follow-up of four groups of patients with chronic tension-type headache [54] treated with anti-depressant medication or placebo with or without stress-management therapy, showed that the placebo had a non-different effect compared to the anti-depressant medication or stress-management therapy given alone on headache activity in the sub-group with initial low CTTH severity and on disability in the sub-group without initial mood and anxiety disorders [56]. This part of the data raises the issue of the placebo efficacy in CTTH of low severity.

The effects of acupuncture in migraine prevention have been evaluated by one meta-analysis [57]: true acupuncture was not superior to sham acupuncture, but is superior to no treatment up to 4 months after treatment (effect size 0.44 SD). In tension type headache, two meta-analysis [58, 59] revealed a small advantage of true acupuncture over sham acupuncture, in fact linked to one heavy positive study [60]. One have to conclude to a lack of specific effect of acupuncture on migraine and to a questionable specific effect of this procedure on tension-type headache. Acupuncture seems to act mostly through a high placebo and non-specific psychological effect.

A cervical pain trial meta-analysis studying manual therapy [61] found that manipulation (high velocity low amplitude) and mobilization produce similar effects on pain and are not better at short- and intermediate-term than controls for pain relief. Consequently, no specific and no significant placebo effects have been demonstrated. However, interpersonal touch has a major impact in our everyday social interactions [62], and has been used as a therapy since the dawn of humanity. Touch therapy is more or less codified (healing touch, therapeutic touch, Reiki) and is consistently associated with a special surrounding that can be considered as having a “non-specific” psychological influence. A meta-analysis of studies conducted on touch therapy for pain [63] includes only one inconclusive study [64] on tension type headache. Therefore, no conclusion about the specific effect of touch on headache can be drawn.

An interesting three arm study [49] on chronic headache sufferers treated by soft manual therapy with relaxation (Trager’s technique), controlled “attentional” visits, or no treatment shows a higher improvement of quality of life in the two treated groups compared to the no-treatment group. Consequently, no specific effect can be concluded from this open study. The improvement in the two groups may be due to the psychological non-specific effect.

Interestingly, the beneficial effect of sham acupuncture on headache has been proposed as a model of ritual healing by touch [65], which provides one way to explain the powerful efficacy of sham acupuncture when compared to no-treatment.

A comprehensive efficacy review of biofeedback (BFB) [66] concluded that true BFB is not significantly superior to sham BFB in migraine (effect size 0.25, confidence interval 95% 0.49–0.00) but did show a small advantage of true BIB over sham in tension-type headaches (effect size 0.50, confidence interval 95% 0.26–0.75). In both conditions, BFB is superior to the waiting list. Thus, BFB seems to have a specific beneficial effect on tension type headache. The superiority of pseudo BFB on the waiting list may be due to the additive effect of the non-specific psychological effect and the placebo effect of BFB.

In children, an interesting three arm study [48] (warming BFB associated with cognitive stress management therapy, pseudo BFB associated with an attention therapy, waiting-list) did not find evidence for significant inter-treatment difference and cannot conclude to a specific effect.

A review of the studies of behavioral treatment of headache [67] reports a 35–55% improvement but also emphasizes many methodological imperfections, including selection bias, credibility of the control procedure, and lack of reproducibility of the results. In addition, most of these studies were performed more than 30 years ago, and a control waiting list group was not reported. Among these studies, an interesting one [46] compares four treatments for tension headache (relaxation, relaxation + cognitive therapy, pseudo-meditation, and waiting list) and reports a significantly better improvement of a headache index for the two groups treated with relaxation compared to the pseudo-meditation group. Pseudo-meditation consisted of an equal number of sessions in which subjects were engaged in imaging daily activity without becoming relaxed, and is therefore a control of the relaxation. This study provides evidence for a specific effect of relaxation on tension type headache prophylaxis.

### As conclusions from this review on RCT

A specific effect of treatment has been demonstrated by meta-analysis in several situation: (1) oral treatment of migraine, and tension type headache attacks, (2) oral treatment for migraine prevention during usually a 12-week administration, regardless the level of control of the attack and the underlying anxio-depressive state. In tension type headache, a questionable specific effect is also reported for acupuncture and for BFB associated with relaxation, and, by one study for relaxation.

A placebo effect is likely associated with every kind of treatment. However the evidences are only indirect. (1) In migraine attack, the amplitude of the improvement in the placebo arms (about half of that observed in the verum arms, if we disregard the improvement due to the natural course) replaces a demonstration. However if we consider the harder outcome of pain free at 2 h, this placebo effect is only about a fifth of that of verum. (2) In oral prophylaxis of migraine, the meta-analysis of short-term RCT reports also an improvement half of that of verum for placebo-treated patients, which is also an indirect proof of a short-term genuine placebo effect. Interestingly one study suggest a long-term placebo effect in chronic tension type headache in patients with a moderate disability or with a low initial anxio-depressive level, and in migraine patient with an optimal attack control.

Both placebo and non specific psychological effect are likely at the origin of the improvement induced by many procedures (migraine prophylaxis by acupuncture or biofeedback, headache in general for manual therapy, touch and behavioral treatment) on the evidences that for these techniques in these precise conditions, the patents improve though no specific effect has ever been demonstrated. The non- specific psychological effects of these complex procedures in headache treatments refer to the “common factors” shared by the various modalities of psychotherapies (see review in [68]

## Towards a theory of the treatment for headache sufferers

Expectation-linked placebo, and non-specific psychological effects, and conditioning, vary according clinical situations and psychological context of the patients.

### Variability of expectation linked placebo and conditioning effects (see also review [69])

The placebo effect linked to expectation is likely low in RCT as this situation does not favor a full effect of suggestion because of the formality of the inclusion, announcement of the side-effects and the known eventuality of receiving a non-active drug. Conversely, suggestions in clinical daily practice may be of great importance: a positive enthusiastic announcement of a beneficial effect will certainly have a better therapeutic effect than a restrictive announcement putting forward side-effects. One can formulate as a reasonable working hypothesis that the efficacy of some charismatic healers or shamans may be associated to massive μ-opioidergic/D2D3-dopaminergic mobilization. Furthermore, in prophylactic repetitive treatment, which is equivalent to a ritual, the effects of suggestion and conditioning are intermingled, likely reenforced by a long delay of occurrence, and a good control of the first attack [56]. Consequently, the more a prophylactic treatment worked at its beginning, the more it will continue working.

### Influence of the psychological and psychopathological context

**Fig. 1:**
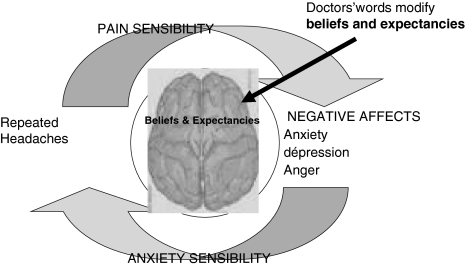
Repeated headaches induce negative affects according to an individual dimension of “pain sensibility”. Negative affects increase repeated headaches according to an individual dimension of “anxiety sensitivity”. Both are modulated, as placebo/nocebo effects by beliefs and expectancies strongly influenced by doctors’ words

Repeated headaches induce negative affects with negative cognitive, affective (pain fear) and physiologic consequences, according to an individual dimension of “pain sensibility”. In return, negative effects can induce attacks, increase their intensity and the subsequent disability according to an individual “anxiety sensitivity” [70, 71] (Table 1; Fig. 1). Both “pain sensibility” and “anxiety sensibility” depend on beliefs, as placebo/nocebo phenomena depend on expectations. In daily clinical practice, the announcements of doctors strongly influence the expectations and beliefs of patients, which then influence the amplitude of placebo effect and of the pain and anxiety sensibilities, and are therefore a major determinant for a therapeutic success. There is an increase in psychopathological co-morbidity in chronic migraine and tension type headache [72, 73]. Consequently, any repetitive treatment, even a placebo, acting on this dimension may modify the natural course of the disease [74]. According to Frank [68], this beneficial effect may be due to the reduction of the “demoralization”, likely presented by the headache sufferers.

**Table 1:** Components of the therapeutic outcome of headache patients

1. Factors directly linked to the treatment
Image of the treatment, announce, expectancy
Repetition of the procedure and conditioning
Touch?
Specific action on headache pathophysiology
2. Factors non directly linked to the treatment
Non-specific psychological support
Words of doctors modifying the beliefs
3. Factors modifying the course of the underlying disease
Optimized attack control
Effect on the concomitant psychopathology

## Conclusion

Headache is the last phase of activation of neuronal networks and can be powerfully controlled by analgesic and psychological systems. Suggestion from the outside, internal beliefs and expectations, and psycho-pathological context are deeply influenced by any therapeutic proposition. Inter-individual variations of these factors are potentially highly important. Consequently, when a patient trusts in a procedure, one has to accept the fact that this procedure is effective for him and produces corresponding biological consequences. Specific treatments act effectively on the common final mechanisms of migraine or tension headache in combination with the other non-specific factor.
